# Human Equilibrative Nucleoside Transporter 1 (hENT1) in Pancreatic Adenocarcinoma: Towards Individualized Treatment Decisions 

**DOI:** 10.3390/cancers2042044

**Published:** 2010-12-02

**Authors:** Jennifer L. Spratlin, John R. Mackey

**Affiliations:** Department of Medicine, Cross Cancer Institute, University of Alberta, 11560 University Avenue, Edmonton, Alberta T6G 1Z2, Canada

**Keywords:** nucleoside transporters, hENT1, pancreatic adenocarcinoma, predictive tool, individualized medicine

## Abstract

Pancreatic cancer is one of the most lethal cancers, where curative surgical resections are rare and less than 5% of patients experience long-term survival. Despite numerous clinical trials, improvements in the systemic treatment of this disease have been limited. Gemcitabine, a nucleoside analogue, is still considered the standard of care chemotherapy for most patients in the advanced disease setting. To exert its cytotoxic effects, gemcitabine must enter cells via nucleoside transporters, most notably human equilibrative nucleoside transporter 1 (hENT1). Increasingly strong evidence suggests hENT1 is a prognostic biomarker in gemcitabine-treated pancreatic cancer, and may well be a predictive biomarker of gemcitabine efficacy. In this review, we synthesize the literature surrounding hENT1 in pancreatic cancer, identify the key outstanding questions, and suggest strategies to prospectively evaluate the clinical utility of hENT1 in future clinical studies.

## 1. Introduction

Pancreatic cancer is estimated to be the tenth most commonly diagnosed cancer and fourth leading cause of cancer related death in 2010 [1]. At diagnosis, approximately 80% of patients have locally advanced, unresectable, or metastatic disease [2]. For those patients with cancers amenable to curative resection, only 5% are long-term survivors [2]. Pancreatic cancer clinical trials have had few successes over the last decade. Gemcitabine remains the standard of care for the first-line treatment of incurable disease for most patients based on the pivotal paper from 1997 demonstrating a 23.8% clinical benefit and modest improvement in overall survival (OS) of 1.24 months over 5-fluorouracil (5FU) [3]. More recent data has indicated oxaliplatin based regimens have a role in both the first- and second-line setting of advanced disease though final publication of these trials are pending. When compared to gemcitabine, the combination of oxaliplatin, irinotecan, and 5FU prolongs progression free survival (PFS) and OS from 3.3 to 6.4 months and 6.8 to 11.1 months, respectively, in treatment naïve patients [4]. Additionally, the CONKO-003 study reported oxaliplatin administered with 24 hours of infusional 5FU had an improvement in PFS and at least a two month OS benefit in gemcitabine refractory pancreatic cancer [5]. Other cytotoxic agents have proven largely ineffective, although many expect gemcitabine with abraxane (nab-paclitaxel) to outperform gemcitabine in a pivotal randomized study currently still accruing patients (NCT00844649).

Unlike advanced colorectal and hepatocellular malignancies, biologic agents have not had success in the treatment of advanced pancreatic cancer. Though the addition of erlotinib to gemcitabine is the only targeted agent to demonstrate statistical significance in a first-line phase III randomized setting, the 0.33 month advantage in median OS and 6% benefit in one-year survival is not generally accepted to be clinically relevant; it is nonetheless an approved agent for use in advanced disease [6]. Other directed therapies, including cetuximab, bevacizumab, sunitinib, sorafenib, the mammalian target of rapamycin pathway inhibitor everolimus, and two matrix metalloproteinase inhibitors, have shown no survival advantage in this setting [7,8,9,10,11,12,13]. Furthermore, combined inhibition of the epidermal growth factor and vascular endothelial growth factor pathways is not efficacious [14,15].

With these negative trial results, gemcitabine remains the reference agent in most clinical trials in advanced pancreatic cancer. Due to the singular importance of this drug in pancreas cancer, significant efforts have gone into finding ways to improve its therapeutic ratio (the relationship between efficacy and toxicity). While pharmacokinetic studies have not changed the current paradigm of “same dose, same schedule, same drug” for everyone, tumor biomarkers are beginning to show promise in the selection of individuals for gemcitabine therapy [16,17]. Among the biomarkers of potential clinical utility, the most promising data is emerging from studies of the human equilibrative nucleoside transporter 1 (hENT1). Below, we review the biology of hENT1, and its relationship to gemcitabine efficacy in the context of treatment of pancreatic adenocarcinoma. Finally, we explore the remaining questions and study designs required to fully validate hENT1 as a predictive marker, capable of wide-scale use to individualize therapy decisions for patients with pancreatic cancer.

## 2. Gemcitabine Transport and Metabolism

**Figure 1:**
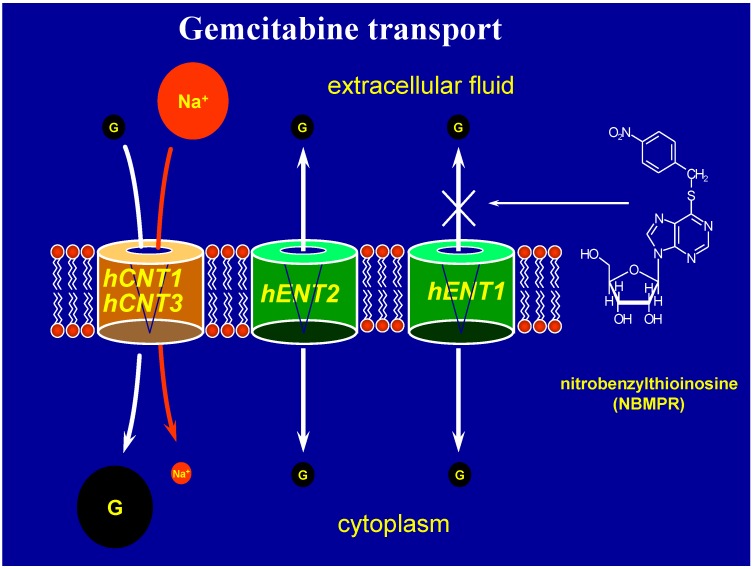
Gemcitabine transport. Abbreviations: Na^+^, sodium; hCNT, human concentrative nucleoside transporter; hENT, human equilibrative nucleoside transporter.

Gemcitabine (2’,2’-difluorodeoxycytidine) is a pyrimidine nucleoside analogue. It exerts its cytotoxic effect intracellularly and has activity against a number of different solid tumors, including pancreatic, breast, lung, and bladder cancers [18]. As gemcitabine is strongly hydrophilic, passive diffusion through the hydrophobic cellular plasma membrane lipid bilayer is slow. In order to efficiently enter cells, gemcitabine requires physiologic nucleoside transporter proteins to cross the plasma membrane [19]. These transporter proteins fall into two categories, equilibrative transporters and concentrative transporters (Figure 1) [19]. The bi-directional human equilibrative nucleoside transporters (hENT) are found in most cell types, and both hENT1 and hENT2 are capable of mediating gemcitabine uptake in the direction of the concentration gradient [20]. The hENT proteins are transmembrane glycoproteins that localize to the plasma membrane. They are functionally distinguished by their ability to be inhibited by nitrobenzylmercaptopurine ribonucleoside (NBMPR), with hENT1 sensitivity in the nanomolar range compared to the relative resistance to NBMPR inhibition exhibited by hENT2. Two identified concentrative nucleoside/sodium co-transporters in humans, human concentrative nucleoside transporter 1 (hCNT1), and hCNT3, are able to bring gemcitabine into a cell, and by virtue of the energy supplied by the physiological sodium-gradient, can do so against the concentration gradient of the substrate [20]. While hCNT3 has the broadest tissue expression, it is also the least selective of the concentrative transporters, accepting both pyrimidine and purine nucleosides, in contrast to hCNT1 and hCNT2 which accept pyrimidine and purine nucleosides, respectively, more favorably [20,21,22].

Gemcitabine cellular uptake is primarily mediated by hENT1, which can be identified at varying levels in all human tissues. While the other transporters (hENT2, hCNT1, and hCNT3) mediate gemcitabine uptake in laboratory conditions [21,22,23,24,25], their contributions to total cellular gemcitabine uptake remain relatively minor, and can only be readily detected when NBMPR is used to inhibit hENT1 activity. Furthermore, *in vitro* data demonstrate cytotoxic nucleoside resistance with hENT1 deficiency [24,26,27,28,29]. Given the short infusion times of gemcitabine (30 minutes) and the short serum half-life of gemcitabine, due to its rapid metabolism and excretion as non-toxic metabolites, it follows that cells with low hENT1 protein abundance might be clinically resistant to gemcitabine.

**Figure 2:**
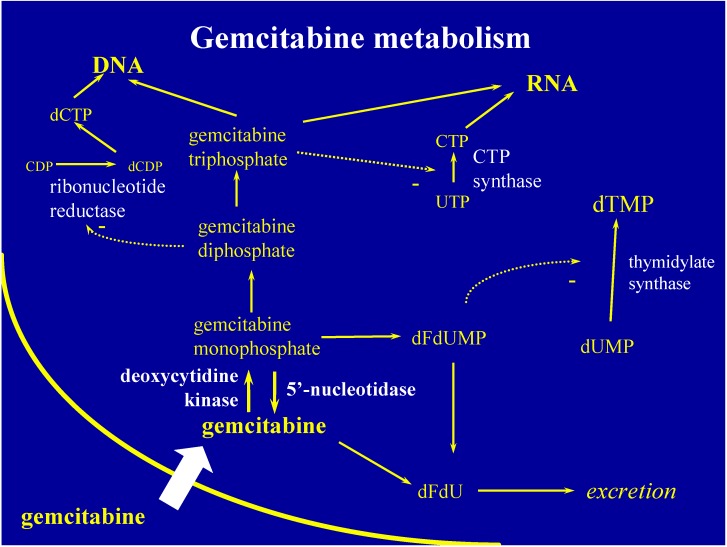
Gemcitabine metabolism. Abbreviations: DNA, deoxyribonucleic acid ; CDP, cytidine diphosphate; dCDP, deoxycytidine diphosphate; dCTP, deoxycytidine triphosphate; gemcitabine, triphosphate form; dFdU, 2’,2’-difluorodeoxyuridine; dFdUMP, gemcitabine, monophosphate form; dUMP, deoxyuridine monophosphate; dTMP, deoxythymidine monophosphate; UTP, uridine-5'-triphosphate; CTP, cytidine triphosphate; RNA, ribonucleic acid.

Once within the cell, nucleotide kinases phosphorylate gemcitabine to gemcitabine monophophate and then sequentially to its active metabolites, gemcitabine diphosphate and gemcitabine triphosphate. The first phosphorylation step by deoxycytidine kinase is the rate limiting step (Figure 2). Once in triphosphate form, gemcitabine is incorporated into cellular DNA and protected from repair by base pair excision with the addition of another natural nucleotide [30,31]. Gemcitabine is self-potentiating and in addition to its masked chain termination of DNA, gemcitabine has other mechanisms of cancer control which include induction of apoptosis by gemcitabine monophosphate, blocking *de novo* DNA synthesis by gemcitabine diphosphate, and lowering the pool of opposing deoxycytidine triphosphate by gemcitabine triphosphate [30,32,33].

## 3. Evidence for the Use of hENT1 as a Biomarker in Pancreatic Cancer

**Figure 3:**
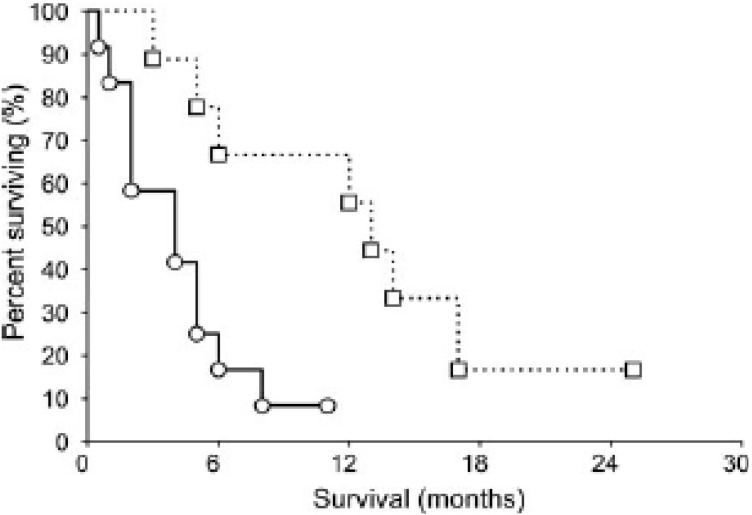
Kaplan-Meier estimate of survival in gemcitabine-treated pancreatic cancer patients. Patients for whom all adenocarcinoma cells had detectable hENT1 (□ and *dashed line*) had significantly longer survival than those patients with heterogeneous areas of adenocarcinoma cells lacking hENT1 (○ and *continuous line*; median survival 13 *versus* four months; P = 0.01). Reproduced with permission from [34].

The first study exploring relationships between hENT1 and gemcitabine efficacy was published in 2004 [34]. We studied the relative abundance of the hENT1 protein, as measured by immunohistochemistry of pancreatic adenocarcinoma biopsies, in a population of patients who received palliative gemcitabine chemotherapy for advanced disease. We described a significant median survival difference (13 months *versus* four months; p = 0.01) when those patients with uniformly detectable hENT1 immunohistochemical (IHC) staining were compared to those who had 10–100% of malignant cells without staining (Figure 3). Although this study was limited by its retrospective evaluation and relatively small number of patients, it set the stage for pre-clinical studies evaluating hENT1 deficiency as a gemcitabine resistance mechanism, and clinical evaluation of hENT1 as a potential predictive biomarker for individualization of gemcitabine therapy.

Pre-clinically, with evaluation of both pancreatic and biliary tract carcinomas, hENT1 is strongly related to gemcitabine chemosensitivity, transport and intracellular gemcitabine accumulation [35,36]. Pancreatic (MIAPaCa2, AsPC1, and BxPC3), gall bladder (OCUG-1), and cholangiocarcinoma (HuCCT1) cell lines treated with gemcitabine were evaluated for mRNA hENT1 levels by quantitative reverse transcription polymerase chain reaction (RT-PCR). hENT1 mRNA levels correlated with the ability of gemcitabine to inhibit growth of these cell lines as determined by inhibitory concentration 50 (IC_50_) levels, indicating higher levels of hENT1 within cell lines is directly associated with chemosensitivity [35].

When evaluated as a prognostic factor for overall survival, disease-free survival, and time to disease progression, pancreatic cancer hENT1 RNA expression correlated with clinical outcomes [37]. Transcriptional analysis of hENT1 by RT-PCR in 102 laser micro-dissected pancreatic cancer specimens demonstrated a three-fold improvement in median overall survival, from 8.5 to 25.7 months, in tumors with higher *versus* lower levels of hENT1 expression [37]. Using immunohistochemistry techniques, hENT1 expression has also been evaluated and correlated with survival in 45 patients with curative intent resection of their pancreatic adenocarcinomas who went on to have post-operative adjuvant chemoradiation [38]. Those patients with high compared to low hENT1 expression had significantly longer OS (not yet reached *versus* 13.3 months (p = 0.0001)) and three-year survival of 68.4% *versus* 19.2% (p = 0.0007). Similarly, in the hENT1 high expression group, disease-free survival (DFS) was 46.8 *versus* 8.4 months (p = 0.0001) in favor of the high hENT1 expression group [38].

The RTOG 9704 study was a randomized phase III study comparing 5-fluorouracil (5FU) with gemcitabine in addition to chemoradiation as adjuvant therapy in resected pancreatic adenocarcinoma [39]. Results from this 451 patient study demonstrated the addition of gemcitabine to adjuvant 5FU-based chemoradiation was associated with a trend in benefit in OS. A retrospective translational research study evaluated tumors from 229 of the patients treated on the RTOG 9704 protocol and tested tissue microarray slides for hENT1 protein abundance [40]. hENT1 protein detection in tumor tissue was independently associated with improved DFS and OS compared to those samples without hENT1 expression; this effect was seen only in the gemcitabine-treated group, and not the 5FU-treated group. This qualitative difference suggests that hENT1 assessment is not only prognostic in a gemcitabine treated population, but has utility as a predictive biomarker for gemcitabine efficacy.

Pharmacogenomic studies of hENT1 have not clearly defined the relevance of the inter-individual sequence variation in the gene encoding the hENT1 protein (called SLC29A1). Although single nucleotide polymorphisms (SNPs) in hENT1 have been identified, none have demonstrated functional consequences in rate of drug uptake or transport [41,42,43]. However, a recent study of 154 patients treated with neoadjuvant gemcitabine suggested that a combined assessment of six SNPs, including the hENT1 T-549C allele and hENT1 C913T allele, did relate to overall survival [44]. While multiple alternatively spliced variants encoding hENT1 have been identified, they have not been shown to have clinical relevance.

## 4. Conclusions and Recommendations

Treatment options available to pancreatic cancer patients remain limited, and few meaningful improvements have been made in the treatment of this disease over the last 30 years. Novel biologic agents have failed categorically—the suite of negative pancreatic cancer trials evaluating cytotoxic and biologic agents, either alone or in combinations, leaves gemcitabine monotherapy as the current standard of care for all but the very best of performance status patients. A thoughtful editorial recently published in the *Journal of Clinical Oncology* has outlined the trials and tribulations of pancreatic cancer research and has suggested modifications to improve clinical trial design and outcomes including: consideration of randomized phase II designs, better patient selection and stratification, questioning gemcitabine as the backbone treatment, better and quicker access to completed trial results, and the identification of surrogate endpoints similar to three year DFS in metastatic colon cancer [45].

Unlike breast cancer, where the presence or absence of the estrogen receptor routinely guides endocrine treatment decisions, a similarly validated biomarker is not yet available for pancreatic cancer treatment decisions. Among pancreas cancer candidate biomarkers, though others are being investigated and have some potential value, hENT1 evaluation has the strongest pre-clinical mechanistic support, and the strongest clinical dataset to suggest a meaningful role as a predictive marker with which to guide treatment decisions [44,46]. However, several questions remain, including i) what is the best target to measure, hENT1 RNA expression or protein abundance; ii) what is the optimal cutpoint to dichotomize populations as gemcitabine sensitive or gemcitabine resistant; iii) how important are preanalytic variables (time to tissue fixation, type of fixation, long-term stability of the epitope) in the performance of the IHC assay; iv) will a robust, quality controlled and regulatory approved standardized assay be available; v) is the hENT1 status of primary tumor concordant with metastases from the same individual; and vi) can hENT1 immunocytochemistry, performed on needle aspirates, provide useful predictive data. Furthermore, confirmation of the predictive ability of hENT1 to distinguish gemcitabine sensitive from gemcitabine insensitive disease will require validation in another large study in which patients are randomized to receive gemcitabine, or not. While additional retrospective analyses of completed studies are underway, the experiment to unequivocally validate such a predictive marker requires a prospective design in which pretreatment hENT1 status is used to stratify patients prior to randomization to gemcitabine based treatment, or to non-gemcitabine based treatment. Such a study would provide the highest level of confidence in this approach, and would, if positive, vault gemcitabine into the select few anticancer agents for which a truly sensitive population can be rationally treated. By this “molecular triage”, the risk:benefit ratio of gemcitabine therapy for pancreatic cancer could be meaningfully improved.

## References

[1] Jemal A., Siegel R., Xu J., Ward E. (2010). Cancer Statistics, 2010. CA Cancer J. Clin..

[2] Yeo T.P., Hruban R.H., Leach S.D., Wilentz R.E., Sohn T.A., Kern S.E., Iacobuzio-Donahue C.A., Maitra A., Goggins M., Canto M.I. (2002). Pancreatic cancer. Curr. Probl. Cancer.

[3] Burris H.A., Moore M.J., Andersen J., Green M.R., Rothenberg M.L., Modiano M.R., Cripps M.C., Portenoy R.K., Storniolo A.M., Tarassoff P. (1997). Improvements in Survival and Clinical Benefit with Gemcitabine as First-Line Therapy for Patients with Advanced Pancreas Cancer: A Randomized Trial. J. Clin. Oncol..

[4] Conroy T., Desseigne F., Ychou M., Ducreux M., Bouche O., Guimbaud R., Becouarn Y., Montoto-Grillot C., Gourgou-Bourgade S., Adenis A. (2010). Randomized Phase III Trial Comparing FOLFIRINOX (F: 5FU/leucovorin [LV], irinotecan [I], and oxaliplatin [O]) versus Gemcitabine (G) as First-Line Treatment for Metastatic Pancreatic Adenocarcinoma (MPA): Preplanned Interim Analysis Results of the PRODIGE 4/ACCORD 11 trial. J. Clin. Oncol..

[5] Pelzer U., Kubica K., Stieler J., Schwaner I., Heil G., Görner M., Mölle M., Hilbig A., Dörken B., Riess H. (2008). A Randomized Trial in Patients with Gemcitabine Refractory Pancreatic Cancer. Final Results of the CONKO 003 Study. J. Clin. Oncol..

[6] Moore M.J., Goldstein D., Hamm J., Figer A., Hecht J.R., Gallinger S., Au H.J., Murawa P., Walde D., Wolff R.A. (2007). Erlotinib plus Gemcitabine Compared with Gemcitabine alone in Patients with Advanced Pancreatic Cancer: A Phase III Trial of the National Cancer Institute of Canada Clinical Trials Group. J. Clin. Oncol..

[7] Philip P.A., Benedetti J., Corless C.L., Wong R., O'Reilly E.M., Flynn P.J., Rowland K.M., Atkins J.N., Mirtsching B.C., Rivkin S.E. (2010). Phase III Study Comparing Gemcitabine plus Cetuximab versus Gmcitabine in Patients with Advanced Pancreatic Adenocarcinoma: Southwest Oncology Group-Directed Intergroup Trial S0205. J. Clin. Oncol..

[8] Kindler H.L., Niedzwiecki D., Hollis D., Sutherland S., Schrag D., Hurwitz H., Innocenti F., Mulcahy M.F., O'Reilly E., Wozniak T.F. (2010). Gemcitabine plus Bevacizumab Compared with Gemcitabine plus Placebo in Patients with Advanced Pancreatic Cancer: Phase III Trial of the Cancer and Leukemia Group B (CALGB 80303). J. Clin. Oncol..

[9] O'Reilly E.M., Niedzwiecki D., Hollis D.R., Bekaii-Saab T.S., Pluard T., Duffy A., Overcash F., Ivy S.P., Goldberg R.M. (2008). A Phase II Trial of Sunitinib (S) in Previously-Treated Pancreas Adenocarcinoma (PAC), CALGB 80603. J. Clin. Oncol..

[10] Wallace J.A., Locker G., Nattam S., Kasza K., Wade-Oliver K., Stadler W.M., Vokes E.E., Kindler H.L. (2007). Sorafenib (S) plus Gemcitabine (G) for Advanced Pancreatic Cancer (PC): A Phase II Trial of the University of Chicago Phase II Consortium. J. Clin. Oncol.

[11] Wolp B.M., Hezel A.F., Abrams T., Blaszkowsky L.S., Meyerhardt J.A., Chan J.A., Enzinger P.C., Allen B., Clark J.W., Ryan D.P. (2009). Oral mTOR Inhibitor Everolimus in Patients with Gemcitabine-Refractory Metastatic Pancreatic Cancer. J. Clin. Oncol..

[12] Bramhall S.R., Schulz J., Nemunaitis J., Brown P.D., Baillet M., Buckels J.A. (2002). A Double-Blind Placebo-Controlled, Randomised Study Comparing Gemcitabine and Marimastat with Gemcitabine and Placebo as First Line Therapy in Patients with Advanced Pancreatic Cancer. Br. J. Cancer.

[13] Moore M.J., Hamm J., Dancey J., Eisenberg P.D., Dagenais M., Fields A., Hagan K., Greenberg B., Colwell B., Zee B. (2003). Comparison of Gemcitabine versus the Matrix Metalloproteinase Inhibitor BAY 12-9566 in Patients with Advanced or Metastatic Adenocarcinoma of the Pancreas: A Phase III Trial of the National Cancer Institute of Canada Clinical Trials Group. J. Clin. Oncol..

[14] Kindler H.L., Gangadhar T., Karrison T., Hochster H.S., Moore M.J., Micetich K., Sun W., Catenacci D.V., Stadler W.M., Vokes E.E. (2008). Final Analysis of a Randomized Phase II Study of Bevacizumab (B) and Gemcitabine (G) plus Cetuximab (C) or Erlotinib (E) in Patients (pts) with Advanced Pancreatic Cancer (PC). J. Clin. Oncol..

[15] Vervenn W., Bennouna J., Humblet Y., Gill S., Moore M.J., Laethem J.V., Shang A., Cosaert J., Verslype C., van Cutsem E. (2008). A Randomized Double-Blind Placebo (P) Controlled Multicenter Phase III Trial to Evaluate the Efficacy and Safety of Adding Bevacizumab (B) to Erlotinib (E) and Gemcitabine (G) in Patients (pts) with Metastatic Pancreatic Cancer. J. Clin. Oncol..

[16] Tanaka M., Javle M., Dong X., Eng C., Abbruzzese J.L., Li D. (2010). Gemcitabine Metabolic and Transporter gene Polymorphisms are Associated with Drug Toxicity and Efficacy in Patients with Locally Advanced Pancreatic Cancer. Cancer.

[17] Tempero M., Plunkett W., Ruiz Van Haperen V., Hainsworth J., Hochster H., Lenzi R., Abbruzzese J. (2003). Randomized Phase II comparison Of Dose-Intense Gemcitabine: Thirty-minute Infusion and fixed Dose rate Infusion in Patients with Pancreatic Adenocarcinoma. J. Clin. Oncol..

[18] Galmarini C.M., Mackey J.R., Dumontet C. (2001). Nucleoside Analogues: Mechanisms of Drug Resistance and Reversal Strategies. Leukemia.

[19] Mackey J.R., Baldwin S.A., Young J.D., Cass C.E. (1998). Nucleoside Transport and its Significance for Anticancer Drug Resistance. Drug Resist. Updat..

[20] Young J.D., Yao S.Y., Sun L., Cass C.E., Baldwin S.A. (2008). Human Equilibrative Nucleoside Transporter (ENT) Family of Nucleoside and Nucleobase Transporter Proteins. Xenobiotica.

[21] Ritzel M.W., Ng A.M., Yao S.Y., Graham K., Loewen S.K., Smith K.M., Ritzel R.G., Mowles D.A., Carpenter P., Chen X.Z. (2001). Molecular Identification and Characterization of Novel Human and Mouse Concentrative Na+-Nucleoside Cotransporter Proteins (hCNT3 and mCNT3) Broadly Selective for Purine and Pyrimidine Nucleosides (system cib). J. Biol. Chem..

[22] Ritzel M.W., Ng A.M., Yao S.Y., Graham K., Loewen S.K., Smith K.M., Hyde R.J., Karpinski E., Cass C.E., Baldwin S.A. (2001). Recent Molecular Advances in Studies of the Concentrative Na+-Dependent Nucleoside Transporter (CNT) Family: Identification and Characterization of Novel Human and Mouse Proteins (hCNT3 and mCNT3) Broadly Selective for Purine and Pyrimidine Nucleosides (system cib). Mol. Membr. Biol..

[23] Mackey J.R., Yao S.Y., Smith K.M., Karpinski E., Baldwin S.A., Cass C.E., Young J.D. (1999). Gemcitabine Transport in Xenopus Oocytes Expressing Recombinant Plasma Membrane Mammalian Nucleoside Transporters. J. Natl. Cancer Inst..

[24] Mackey J.R., Mani R.S., Selner M., Mowles D., Young J.D., Belt J.A., Crawford C.R., Cass C.E. (1998). Functional Nucleoside Transporters are Required for Gemcitabine Influx and Manifestation of Toxicity in Cancer Cell Lines. Cancer Res..

[25] Garcia-Manteiga J., Molina-Arcas M., Casado F.J., Mazo A., Pastor-Anglada M. (2003). Nucleoside Transporter Profiles in Human Pancreatic Cancer Cells: Role of hCNT1 in 2', 2'-Difluorodeoxycytidine- Induced Cytotoxicity. Clin. Cancer Res..

[26] Wiley J.S., Jones S.P., Sawyer W.H., Paterson A.R. (1982). Cytosine Arabinoside Influx and Nucleoside Transport Sites in Acute Leukemia. J. Clin. Invest..

[27] Wiley J.S., Jones S.P., Sawyer W.H. (1983). Cytosine Arabinoside Transport by Human Leukaemic Cells. Eur. J. Cancer Clin. Oncol.

[28] Paproski R.J., Wuest M., Jans H.S., Graham K., Gati W.P., McQuarrie S., McEwan A., Mercer J., Young J.D., Cass C.E. (2010). Biodistribution and Uptake of 3'-Deoxy-3'-Fluorothymidine in ENT1-Knockout Mice and in an ENT1-Knockdown Tumor Model. J. Nucl. Med..

[29] Cass C.E., King K.M., Montano J.T., Janowska-Wieczorek A. (1992). A Comparison of the Abilities of Nitrobenzyl Thioinosine, Dilazep, and Dipyridamole to Protect Human Hematopoietic Cells from 7-Deazaadenosine (tubercidin). Cancer Res..

[30] Huang P., Plunkett W. (1995). Induction of Apoptosis by Gemcitabine. Semin. Oncol..

[31] Ruiz van Haperen V.W., Veerma G., Vermorke J.B., Peters G.J. (1993). 2', 2'-Difluoro-deoxycytidine (Gemcitabine) Incorporation into RNA and DNA of Tumour Cell Lines. Biochem. Pharmacol..

[32] Plunkett W., Huang P., Searcy C.E., Gandhi V. (1996). Gemcitabine: Preclinical Pharmacology and Mechanisms of Action. Semin. Oncol..

[33] Heinemann V., Schulz L., Issels R.D., Plunkett W. (1995). Gemcitabine: A Modulator of Intracellular Nucleotide and Deoxynucleotide Metabolism. Semin. Oncol..

[34] Spratlin J., Sangha R., Glubrecht D., Dabbagh L., Young J.D., Dumontet C., Cass C., Lai R., Mackey J.R. (2004). The Absence of Human Equilibrative Nucleoside Transporter 1 is Associated with Reduced Survival in Patients with Gemcitabine-Treated Pancreas Adenocarcinoma. Clin. Cancer Res..

[35] Mori R., Ishikawa T., Ichikawa Y., Taniguchi K., Matsuyama R., Ueda M., Fujii Y., Endo I., Togo S., Danenberg P.V. (2007). Human Equilibrative Nucleoside Transporter 1 is Associated with the Chemosensitivity of Gemcitabine in Human Pancreatic Adenocarcinoma and Biliary Tract carcinoma Cells. Oncol. Rep..

[36] Nakano Y., Tanno S., Koizumi K., Nishikawa T., Nakamura K., Minoguchi M., Izawa T., Mizukami Y., Okumura T., Kohgo Y. (2007). Gemcitabine Chemoresistance and Molecular Markers Associated with Gemcitabine Transport and Metabolism in Human Pancreatic Cancer Cells. Br. J. Cancer.

[37] Giovannett E., Del Tacca M., Mey V., Funel N., Nannizzi S., Ricci S., Orlandini C., Boggi U., Campani D., Del Chiaro M. (2006). Transcription Analysis of Human Equilibrative Nucleoside Transporter-1 Predicts Survival in Pancreas Cancer Patients Treated with Gemcitabine. Cancer Res..

[38] Marechal R., Mackey J.R., Lai R., Demetter P., Peeters M., Polus M., Cass C.E., Young J., Salmon I., Deviere J. (2009). Human Equilibrative Nucleoside Transporter 1 and Human Concentrative Nucleoside Transporter 3 Predict Survival after Adjuvant Gemcitabine Therapy in Resected Pancreatic Adenocarcinoma. Clin. Cancer Res..

[39] Regine W.F., Winter K.A., Abrams R.A, Safran H., Hoffman J.P., Konski A., Benson A.B., Macdonald J.S., Kudrimoti M.R., Fromm M.L. (2008). Fluorouracil vs Gemcitabine Chemotherapy before and after Fluorouracil-Based Chemoradiation Following Resection of Pancreatic Adenocarcinoma: A Randomized Controlled Trial. JAMA.

[40] Farrell J.J., Elsaleh H., Garcia M., Lai R., Ammar A., Regine W.F., Abrams R., Benson  A.B., Macdonald J., Cass C.E. (2009). Human Equilibrative Nucleoside Transporter 1 Levels Predict Response to Gemcitabine in Patients with Pancreatic Cancer. Gastroenterology.

[41] Osato D.H., Huang C.C., Kawamoto M., Johns S.J., Stryke D., Wang J., Ferrin T.E., Herskowitz I., Giacomini K.M. (2003). Functional Characterization in Yeast of Genetic Variants in the Human Equilibrative Nucleoside Transporter, ENT1. Pharmacogenetics.

[42] Kim S.R., Saito Y., Maekawa K., Sugiyama E., Kaniwa N., Ueno H., Okusaka T., Morizane C., Yamamoto N., Iked A.M. (2006). Thirty Novel Genetic Variations in the SLC29A1 Gene Encoding Human Equilibrative Nucleoside Transporter 1 (hENT1). Drug Metab. Pharmacokinet..

[43] Myers S.N., Goyal R.K., Roy J.D., Fairfull L.D., Wilson J.W., Ferrell R.E. (2006). Functional Single Nucleotide Polymorphism Haplotypes in the Human Equilibrative Nucleoside Transporter 1. Pharmacogenet. Genomics.

[44] Okazaki T., Javle M., Tanaka M., Abbruzzese J.L., Li D. (2010). Single Nucleotide Polymorphisms of Gemcitabine Metabolic Genes and Pancreatic Cancer Survival and Drug toxicity. Clin. Cancer Res..

[45] Tabernero J., Macarulla T. (2009). Changing the Paradigm in Conducting Randomized Clinical Studies in Advanced Pancreatic Cancer: An Opportunity for Better Clinical Development. J. Clin. Oncol..

[46] Fujita H., Ohuchida K., Mizumoto K., Itaba S., Ito T., Nakata K., Yu J., Kayashima T., Souzaki R., Tajiri T. (2010). Gene Expression Levels as Predictive Markers of Outcome in Pancreatic Cancer after Gemcitabine-Based Adjuvant Chemotherapy. Neoplasia.

